# Processing and Analysis of Long-Range Scans with an Atomic Force Microscope (AFM) in Combination with Nanopositioning and Nanomeasuring Technology for Defect Detection and Quality Control

**DOI:** 10.3390/s21175862

**Published:** 2021-08-31

**Authors:** Ingo Ortlepp, Jaqueline Stauffenberg, Eberhard Manske

**Affiliations:** Institute of Process Measurement and Sensor Technology, Technische Universität Ilmenau, 98693 Ilmenau, Germany; jaqueline.stauffenberg@tu-ilmenau.de (J.S.); eberhard.manske@tu-ilmenau.de (E.M.)

**Keywords:** long-range AFM scans, planar nanopositioning, nanofabrication machine, active microcantilevers

## Abstract

This paper deals with a planar nanopositioning and -measuring machine, the so-called nanofabrication machine (NFM-100), in combination with a mounted atomic force microscope (AFM). This planar machine has a circular moving range of 100 mm. Due to the possibility of detecting structures in the nanometre range with an atomic force microscope and the large range of motion of the NFM-100, structures can be analysed with high resolution and precision over large areas by combining the two systems, which was not possible before. On the basis of a grating sample, line scans over lengths in the millimetre range are demonstrated on the one hand; on the other hand, the accuracy as well as various evaluation methods are discussed and analysed.

## 1. Introduction

The trend towards increasingly shrinking structures according to Moore’s Law is advancing within various research areas, such as nanophotonics, nanooptics and nanoelectronics [1]. In addition, the semiconductor industry is following this trend, with a simultaneous focus on mass production and cost reduction. This includes reducing the structure size on the one hand and increasing the wafer size on the other hand in order to achieve the highest possible yield [2].

In order to detect the smallest possible structures for surface imaging, scanning probe microscopy methods can be used [3,4]. This also includes atomic force microscopy (AFM), in which the bending of a microcantilever provides information about the topography of the sample to be analysed [5]. Most of these tip-based methods work with high accuracy in ranges of a few micrometres and are accordingly limited in the analysis of large areas.

In this work, an atomic force microscope is utilised, which uses active microcantilevers. These self-sensing and self-actuating microcantilevers can be applied to measure topography. However, it is also possible to use them for scanning probe lithography [6,7]. With this so-called field-emission scanning probe lithography (FESPL), structures with sizes below sub-10 nm can be generated [8,9].

In addition, a planar nanopositioning and measuring machine was developed [10]. This nanofabrication machine (NFM-100) is especially designed for two-dimensional tasks and has a movement range of 100 mm in diameter. The use of laser interferometers makes it possible to measure the position of the machine table with nanometre precision and a resolution of 5 pm [11].

In principle, the NFM-100 can be equipped with various tools for measuring structures in the nanometre range, such as AFM or laser focus sensors, as well as for micro- and nanostructuring, such as FESPL, direct laser writing (DLW) or nanoimprint lithography (NIL) [12]. At present, the NFM-100 is equipped with an AFM/FESPL system. The novel combination of a nanopositioning machine, which is specialised for planar applications, and a tip-based measuring and writing system makes it possible to detect the smallest structures below 1 nm over extremely large areas, in contrast to conventional AFM systems, and, above all, to produce nanostructuring with high resolution on wafer sizes of up to 4 inches [13]. In addition, the growing demand for measuring periodic structures over long ranges is increasing, so the NFM-100 in combination with the tip-based system opens up new possibilities for measuring long ranges such as precision grids. On the one hand, this enables quality control as well as the investigation of nanostructures in large working areas, tending towards wafer size. In the future, repair of these structures using various nanomanufacturing processes [12] will be explored.

In this paper, the focus is on AFM measurements over the millimetre range. Using line scans over a length of 50 mm on a periodic grid structure, the compatibility of the two measurement systems described is demonstrated and the accuracy is also examined in more detail using various evaluation methods. Furthermore, a comparison of the measurement results at different measuring speeds and a distinction between the different evaluation methods is made.

## 2. Configuration of the Measurement Set-Up Used

The state of the art for measuring periodic structures is the use of optical measuring methods, based on the diffraction of a laser beam and the subsequent determination of the refractive angle [14]. However, these methods have a poor lateral resolution because of the diameter of the laser beam. This is due to the integration of the structural properties over the laser spot size and results in measuring the mean pitch for the investigated area. The approach proposed in this paper has a significantly better lateral resolution in the nanometre range based on the high-resolution AFM system.

### 2.1. Tip-Based Measuring System with Active Microcantilevers

**Figure 1:**
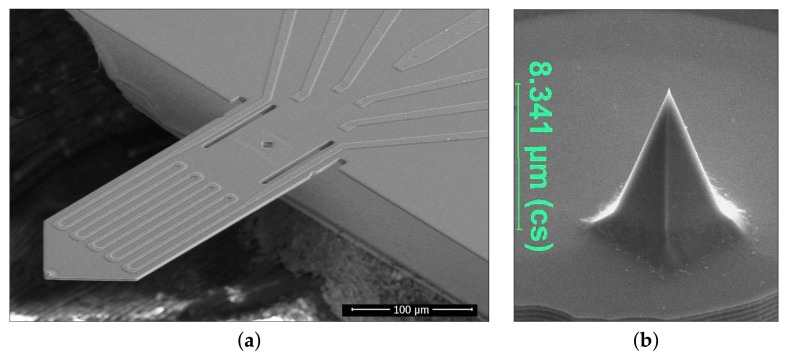
Magnification of the used self-sensing and self-actuated microcantilevers (courtesy of nano analytik GmbH, Ilmenau, Germany [16,17]). (**a**) SEM side-view image of the probe with integrated piezoresistive read-out and thermomechanical actuation (active cantilever). A sharp silicon tip (**b**) at the end of the cantilever ensures advanced AFM imaging and SPL capabilities [13]. These microcantilevers have dimensions of around 350 µm in length, 120 µm in width and 5 µm in thickness [15].

The development of tip-based cantilevers has progressed steadily in recent decades. There are different ways to excite the microcantilever as well as to choose the read-out of the bending of the cantilever. In the work presented here, active microcantilevers are used. As can be identified in Figure 1, the microcantilevers use a piezoresistive read-out to detect the deflection of the beam. According to the piezoresistive effect, a mechanical tension induced in resistors leads to a change in resistivity. By applying this in the form of a Wheatstone resistance measuring bridge, an improvement in performance in terms of temperature stability and deflection sensitivity could be achieved. Furthermore, a significantly improved signal-to-noise ratio was reached through optimised resistor technology [15].

Moreover, as can be seen in Figure 1, the active microcantilever uses a thermomechanical actuator to excite the oscillation. This thermomechanical actuator works according to the bimorph effect and consists of two different layers with various thermal expansion coefficients. In this case, silicon and aluminium are used as layers. The mismatch of the thermal resistance coefficients leads to a higher actuator efficiency [15]. If a periodic signal is now applied to the meander actuator, this leads to an oscillation of the cantilever due to a periodic temperature change [16]. The change in bending can therefore be detected by the resistance bridge at the clamping point.

### 2.2. Nanopositioning and Nanomeasuring Machine

Usually, tip-based systems are characterised by high resolution when analysing the roughness or searching for defects in a given surface, but the scanning area is limited to a range of 100 μm × 100 μm in conventionally available systems. To overcome this limitation, the combination with nanopositioning and nanomeasuring machines represents a novel option. For more than 20 years now, the Institute for Process Measurement and Sensor Technology has been involved in research into nanopositioning systems. The focus here is on measurement and positioning on the basis of laser interferometry according to the Abbe comparator principle in extended three-dimensional working areas [18]. Based on this expertise, two different machines have been developed: the nanomeasureming machine NMM-1, with a working range of 25 mm × 25 mm × 5 mm [19], and the nanopositioning machine NPMM-200, with a range of motion of 200 mm × 200 mm × 25 mm [20]. These machines work according to the scanning stage principle and can move the machine table in three dimensions. In contrast, the machine used in this work has a planar driving system. The adjustment to height variations is to be made by the installed measuring or writing system. However, the aim of the NFM-100 was to achieve greater stability in the z-direction, which is necessary for a sensitive tip-based system.

**Figure 2:**
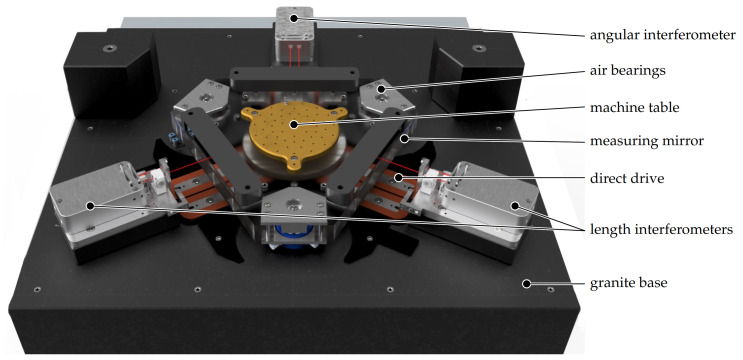
CADmodel of the Nanofabrication Machine (NFM-100). The AFM measuring system is mounted directly above the center of the machine table but is not depicted here for better visibility of the NFM-100.

Due to its high stability in the z-direction, the NFM-100 represents a special platform for investigating new nanomeasuring technologies and alternative lithography processes on extended macroscopic working ranges. This nanopositioning machine uses a planar direct drive system with a range of motion of 100 mm in diameter. A model of its design is depicted in Figure 2. The entire setup including the tip-based system is presented in [13].

A large granite block serves as the basis of the machine [21]. The topside is finished with a flatness of below 1 μm and serves as the guiding surface for the air bearings of the machine slider.

These have the property of enabling high repeatability and, above all, stability. In this way, the highest possible accuracy with almost frictionless and jerk-free movement is guaranteed. To perform the movement of the machine table, direct drives in the form of three linear actuators are used. These have the advantage that there are no disturbances in regard to cogging forces during movement, even at low speeds. They are also characterised by high dynamics and repeatability with respect to positioning. The driving systems are arranged in a 3×120° orientation and can apply three independent forces to the slider in the *z* plane. This arrangement offers three degrees of freedom: the movement in *x* and *y* directions as well as the rotation around the *z* axis, when properly controlled. The machine table is equipped with three measuring mirrors for the length measuring systems.

Fibre-coupled interferometers are used to precisely track the position of the machine table. Here, one stabilised He-Ne laser is used to supply the three interferometer heads [10]. Two interferometers are arranged to measure the length in the *x* and *y* directions. A third interferometer is used to measure the rotation around the *z* axis. The laser interferometers represent the only measurement systems for the *x* and *y* axes, and, thus, the laser wavelength serves as the length scale for these coordinates. The AFM only covers the *z*-measuring direction and does not scan in the *x* and *y* directions. To calibrate the laser wavelength, it was compared to a traceable laser, stabilised to an iodine absorption cell. The actual laser wavelength determined in this way is considered in the machine control system. Thus, the movement and measurement in the *x* and *y* directions is carried out with very high accuracy. The measurement in the *z* direction realised by the AFM does not significantly contribute to the pitch measurement as the main information there is located in the *x* and *y* spatial directions. However, the *z* axis of the AFM can be calibrated by, e.g., a step height standard, if necessary. Because of the planar setup, the machine coordinate system is defined by the three measuring mirrors on the machine slider. Thus, the straightness of the *x* and *y* axes is defined by the flatness of these measuring mirrors and the angle between *x* and *y* by the angle between the mirror stripes. As there are no mechanical representations of the axes, there is no coupling between them. The only mechanical representation inside the machine coordinate system is the *z* plane, which is defined by the granite base. As the *z* height is not actively controlled, flatness errors of the granite directly lead to movements of the sample in the *z* direction. Thus, these movements have to be covered by the AFM measuring system. However, from a metrological point of view, *z* movements induced by the machine table are nonhazardous as they can be measured by an optional *z* interferometer and can be compensated in the measuring data afterwards.

## 3. Measurement Strategy

**Figure 3:**
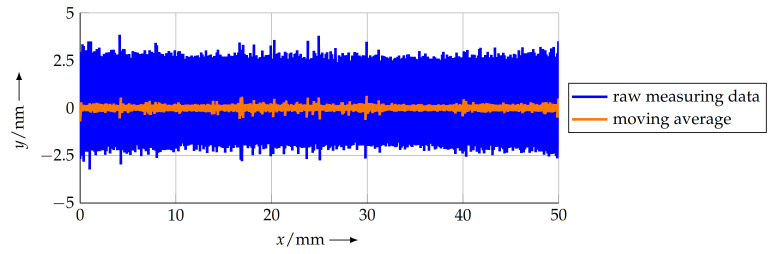
Signals of the *x* and *y* interferometer during linescan with a velocity of 20 μm $s^{-1}$ over a length of 50 mm driven by the machine table of the NFM-100, which shows the deviation of a straight line.

Due to the limited scanning range of conventional tip-based systems, the use of positioning systems is beneficial. Regarding long-range line scans, research results have already been achieved [22,23]. Here, the NMM-1 was used as a nanopositioning system and is accordingly limited to an area of 25 mm × 25 mm × 5 mm. With the newly developed NFM-100, it is possible to measure over ranges up to 100 mm. As mentioned, in [13], first line scans over 50 mm could be demonstrated. The sample used was a LIF 401 length scale from Heidenhain [24] with a nominal pitch of $p_{n}=8\mu m$ and a step height of 200 nm. It is made of glass and is classified as accuracy class 3 μm. In this paper, the sample was used over again for analysis with different methods to evaluate the accuracy of the systems and to explore the ability of the systems for the quality control of the micro- and nanostructures. However, different scan velocities are analysed and evaluated here in terms of measurement accuracy. The line scans were performed with scanning velocities of 5 μm $s^{-1}$, 10 μm $s^{-1}$, 20 μm $s^{-1}$, 30 μm $s^{-1}$, 40 μm $s^{-1}$, and 50 μm $s^{-1}$. For these measurements, the movement in the *x* and *y* directions is realised by the machine table. The movement in the *z* direction is compensated by the tip-based system. The same start and end points were used and driven through the machine table over a total length of 50 mm. The values measured by the interferometers were recorded simultaneously alongside the values measured by the AFM unit. For a velocity of 20 μm $s^{-1}$, the values of the *x* and *y* interferometers are depicted in Figure 3.

The measured values of the interferometers are acquired with a sampling rate of 10 kHz. The standard deviation for a movement of 50 mm in length and a velocity of 20 μm $s^{-1}$ is approximately 825 pm in the y direction. With a moving average of 1000, a standard deviation of 64 pm is achieved (Figure 3). Even for velocities up to 50 μm $s^{-1}$, the standard deviation with respect to the ideal trajectory is below 4 nm.

The sampling rate of the AFM system is time-constant and thus changes with the moving velocity per period; this is summarised in Table 1 for different moving velocities.

## 4. Processing and Analysis of Measurement Data

### 4.1. Sample Misalignment and Sample Deformation

When placing the measuring object on the machine table of the NFM-100, despite elaborate adjustment, the orientation of the object will be slightly rotated with respect to the machine coordinate system. To remove the tilt around the *x* axis and *y* axis from the measuring data, a straight line is fitted into the measurement points via a least squares process. Afterwards, the measuring data are rotated into the *z* plane; thus, distortion (shortening) of the measurement data is avoided, in contrast to simply projecting onto the *z* plane, resulting in a cosine error [25].

Additionally to the imperfect orientation, there is also a deviation from a perfect plane with respect to the line of the measuring object. This is because of the form deviation of the sample due to gravity, inner stress, non-ideal support and so on. However, some evaluation methods described in the following sections rely on a straight scan line and a profile without deformation. To straighten the measuring data, the profile to evaluate has to be isolated from the superposition with the long-wavelength form of the sample. The reconstruction of the straight profile in this project was realised by high-pass filtering of the measurement data. In turn, low-pass filtering was performed to suppress the nominal pitch in the measuring data and calculate the deformation line. Subsequently, this line was subtracted from the data points to achieve effective high-pass filtering and to straighten the profile in this way.

Implementing a low-pass filter for this purpose is possible in various ways. A simple sliding average filter can ideally suppress the nominal pitch, if the length is chosen appropriately to the length of a full period. Besides the nominal pitch, this type of filter will also suppress integer fractions thereof. However, the pitches between this integer fraction will only be poorly suppressed. Thus, this information would afterwards be removed from the measuring data, though it might contain important information on the sample. This is why a low-pass filter according to the Butterworth design [26] was applied. This type of filter has a flat frequency response in the pass band and stop band, ensuring the uniform suppression of pitches in the desired range.

**Figure 4:**
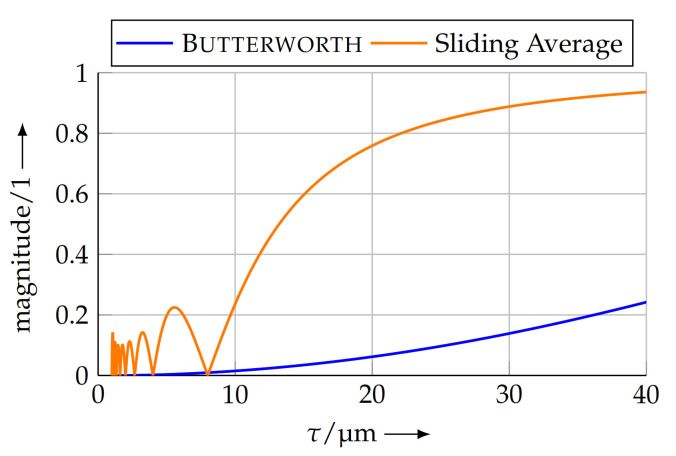
Frequency responses of the sliding average filter with a window length corresponding to $p_{n}=8\mu m$ and a 2nd order Butterworth filter with a cut-off wavelength of $\tau_{c}=10p_{n}=80\mu m$. The sliding average filter offers perfect suppression of integer fractions of the window length, but lacks suppression of the wavelengths between.

The filter configuration was designed as an 2nd order type with a cut-off wavelength of $\tau_{c}=10\;p_{n}$. With this configuration, the nominal pitch is suppressed by a factor of 100. Figure 4 shows a comparison of the frequency responses of the sliding average and the Butterworth filter.

**Figure 5:**
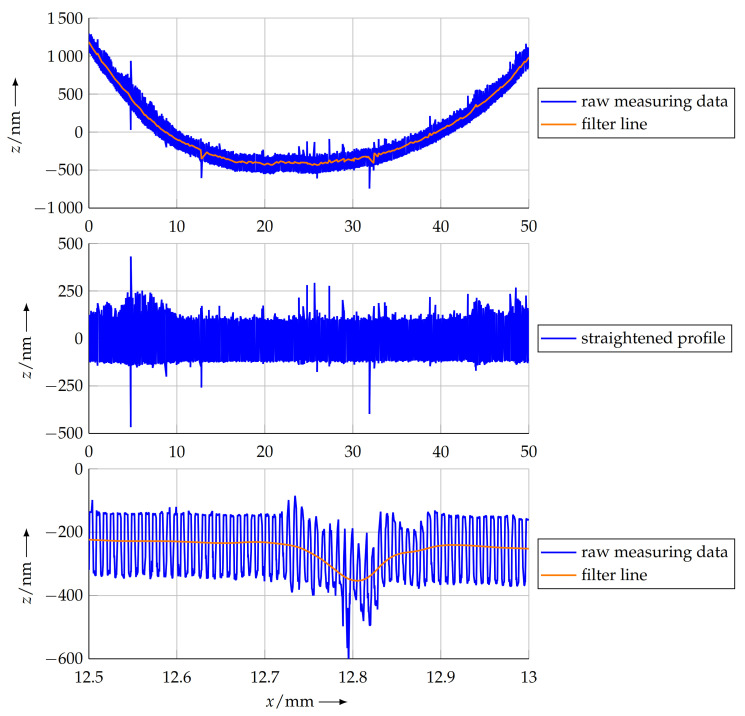
Profiledata of the measured grating sample. The first plot shows the original data over the complete measuring length, rotated to the *z* plane and the filter line, resulting from applying the Butterworth filter described above to the measuring data. The second plot shows the profile data with tilt and bend removed by subtracting the filter line from the measuring data. In the third plot, a detail of the scan line is depicted. Here, it is observable how the filter follows the form deviations of the sample.

When applying this filter to the measuring data, the resulting filter line represents the deviation of the sample from a straight line. This deviation is then subtracted from the measurement data to straighten the profile. This progress is depicted in Figure 5.

### 4.2. Noise in Measurement Data

In general, it is possible to remove noise in the measuring data by low-pass filtering with, e.g., a cut-off wavelength of $\tau_{c}=MM1\;/\;10p_{n}$. In this way, in combination with removing the deformation above, the process would primarily preserve the wavelength of the nominal pitch. This would be of use, for example, for the centre of gravity (COG) method (Section 4.3.1), which gains from a clean profile, so no phantom pitches are detected because of noise, peaks, overshoots and so on. However, distortions of the scanned profile, such as slopes of sidewalls, an asymetric profile (duty factor ≠ 50 ), non-flat top and bottom plateaus and other parameters that might be of interest, could also be suppressed by applying a low-pass filter. When applying the high-pass filter from Section 4.1 alone, only low-frequency errors with much larger wavelength than $p_{n}$ are removed. Thus, the sample profile will be preserved as far as possible. As this results in the least modified measuring data, low-pass filtering was rejected.

### 4.3. Analysis Methods

The preprocessed measurement data are the basis for the subsequent analysis and calculation of the pitch value. In this regard, different evaluation methods are available. A simple approach would be the detection of the intersection point between the mean profile height and the profile line. However, this method is based on a poor statistical base as it only takes into account data points directly around the mean line. For this reason, only evaluation methods using the complete measurement information were used in this project. This includes methods providing information about the local pitch (COG method, Section 4.3.1) as well as methods that calculate the mean pitch over the entire scan length. In this regard, a Fourier transform (Section 4.3.2), an approximation by an ideal profile (Section 4.3.3) and a lock-in method were investigated. Finally, all pitch values calculated by the different methods were compared (Section 4.4).

#### 4.3.1. Centre of Gravity (COG)

**Figure 6:**
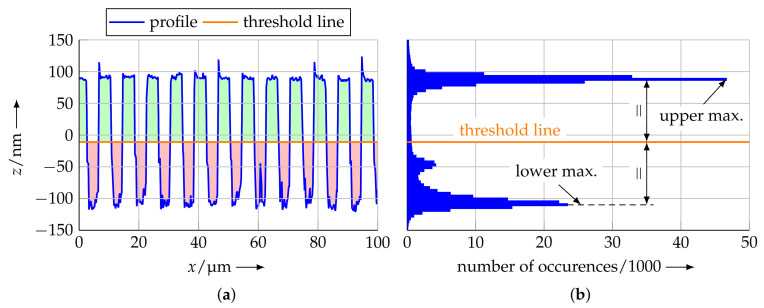
Calculatingthe threshold line from the profile data. A histogram (**b**) is calculated for the measuring points of the profile (**a**, detail) over the complete scan length. In the histogram, the peaks above and below the *x* axis are determined. The threshold line is placed symmetrically between these peaks by calculating their unweighted mean. With the threshold line, the profile can be divided into an upper (green) and a lower (red) part.

The COG method [27] divides the measuring points into two classes and evaluates the area of the profile above and below a threshold line, located approx. in the middle of the profile. To calculate this threshold line, a histogram method is used. In this regard, the histogram of the *z* coordinates of all measurement points is calculated. Because of the plateaus in the profile, there is an accumulation of measuring points in this area. This results in two peaks in the histogram at the according levels. The maxima of the histogram are used to identify these levels and, based on these two values, the threshold line is calculated as the mean of the upper and lower peak in the histogram (Figure 6).

**Figure 7:**
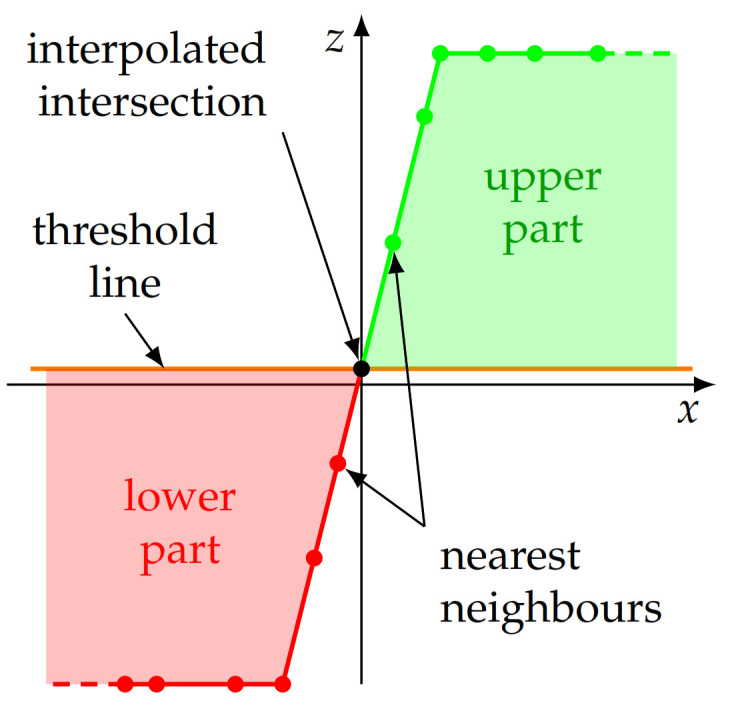
The intersection of the profile line and the threshold line is calculated by linear interpolation between the data points of the profile, located nearest to the threshold line. This additional data point allows more accurate calculation of the area enclosed between profile and threshold line for the subsequent COG calculation.

To calculate the COG of the upper (green in Figure 6) and lower (red in Figure 6) parts of the profile, the exact enclosed area between the according measurement points and the threshold line needs to be known. However, at the transition between an upper and a lower profile section, there is not necessarily a data point, located exactly on the threshold line. When numerically calculating the enclosed area, this would lead to an error. Therefore, the intersection points of the profile line and the threshold line are estimated by linearly interpolating between one data point of the upper and lower section each, located nearest to the threshold line (Figure 7).

**Figure 8:**
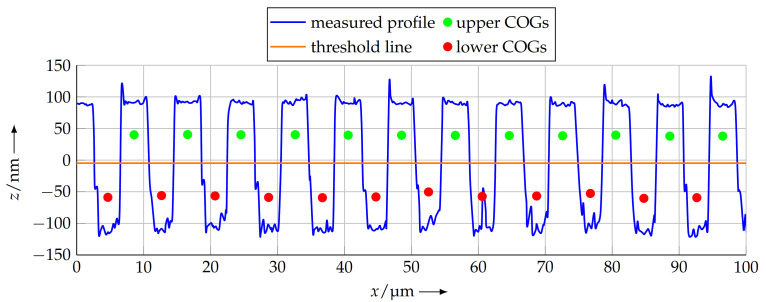
Calculated COGs for the scanned profile (detail). In the *x* direction, the COGs are located at the position where the area of the according profile element is divided into two equal parts. The *z* position of the COGs is not relevant for the pitch calculation and introduced for descriptive purposes only.

After the crossings of the scan line and the threshold line were estimated, the enclosed area was calculated for each profile section above and below the threshold line. This results in twice as many areas as the number of full periods of the profile. For each of these, the *x* position is calculated, at which the area is divided into two equal parts, and this position is used as the COG in the *x* direction. As the *z* position of the COG is not relevant for the determination of the pitch, it is estimated by half of the median of the data points in a single half period for plotting purposes only. Figure 8 shows a detail of the measured profile and the calculated COGs therein.

**Figure 9:**
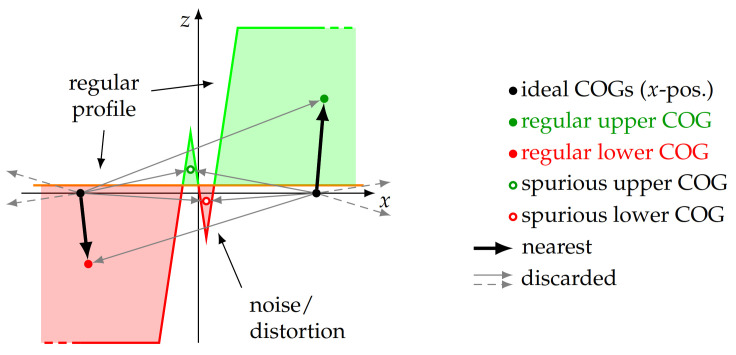
Spurious COGs, caused by additional crossings of the profile line and the threshold line, e.g., because of noise. To eliminate these, a sanity check and a selection of the detected COGs is executed. For this purpose, a synthetic grid of ideally spaced COGs (black dots) is generated and the nearest COG, obtained from measuring data, is determined (black arrows). All other COGs are discarded for this particular ideal COG (grey arrows). Only COGs related to the ideal grid are used for the subsequent calculations.

The calculated *x* positions now represent the COGs of the upper and lower parts of the profile and, thus, the half-pitch of the sample. However, because of distortions in the measurement process, it is possible that additional crossings of the profile line over the threshold line can occur. This in turn results in additional and mistakenly detected profile elements, affecting the pitch calculation. To prevent such distortions, a grid of ideally located COGs with the nominal pitch $p_{n}$ is calculated. For each of these grid points, which are evenly spaced by a distance $p_{n}$, the COG is identified, which is closest to the ideal point. Thus, to each ideal point, a COG based on real measurement data is related. For evaluation of the pitch, now, only the related COGs are used. Unrelated and thus surplus COGs, resulting from erroneous profile detection because of measurement distortions, are discarded. This allows us to sort out invalid detected COGs without affecting the remaining data (Figure 9).

For the remaining COGs, a best fit line in the least squares sense is calculated. As the data consist of upper and lower COGs, the slope of this line represents the half pitch of the grating. The full pitch $p_{\text{COG}}$ of the grating, calculated for a scan velocity of 10 μm $s^{-1}$, is

(1) $$p_{\text{COG}}=8.0038\;\mu m.$$

**Figure 10:**
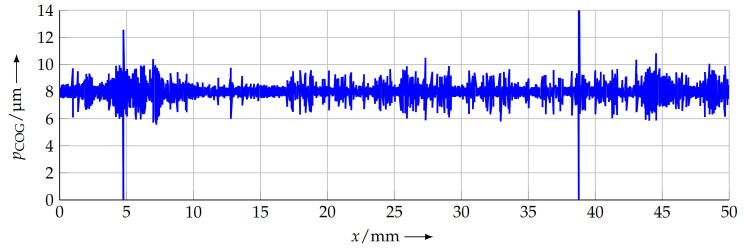
Local pitch of the measured grating. As the COG method allows us to calculate the pitch for every period of the sample, local deviations from the nominal pitch $p_{n}$ can be detected.

As the COG evaluation is based on a per-period calculation, it provides information about the local pitch of the investigated profile. The local pitch $p_{\text{COG},i}$ can be calculated by

(2) $$p_{\text{COG},i}=2\left(x_{\text{COG},i+1}-x_{\text{COG},i}\right),\;i\in N,$$

where $x_{\text{COG},i}$ is the *x* position of the COG for a single profile element (coloured area in Figure 9). Figure 10 shows the pitch value for every sample period over the complete scan line.

As can be seen in Figure 10, the main advantage of the COG method is the high-resolution information about the local pitch. Furthermore, the mean pitch over the complete scan length can be calculated by the described best-fit line. The methods described in the following section only provide pitch information for the complete length to which they are applied.

#### 4.3.2. Fourier Transform

As the measured profile is a periodic structure, it can be analysed for its frequency components. This can be achieved by a Fourier transform, which can calculate the amplitudes of contained spectral components. Here, the spectral component with the maximum amplitude represents the mean frequency over the complete scan length of the measured profile. Of course, the calculated frequency can be converted to the pitch of the grating. Usually, the Fourier transform is carried out by utilising built-in functions of available software. However, a Fast-Fourier-Transform (FFT) is typically used, which is limited in its frequency resolution, depending on the sample rate and the number of data points. Thus, the FFT can only be used for a raw calculation of the mean pitch.

**Figure 11:**
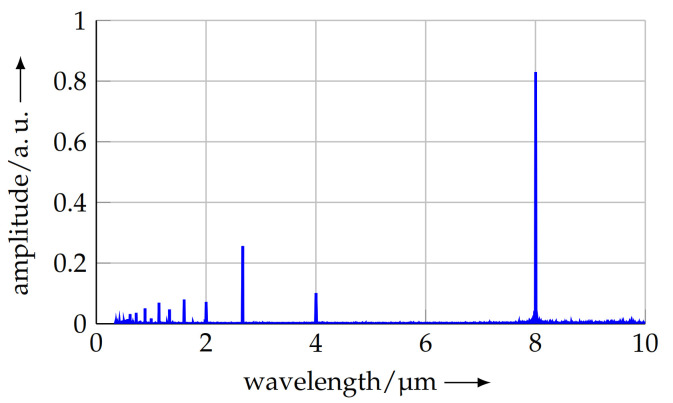
Normalised FFT of the measured profile. The maximum amplitude located at 8 μm represents the mean pitch of the sample grating. Peaks at 4 μm, 2.67 μm, etc., result from the shape of the profile (see text).

The calculation of the FFT relies on equally spaced data points in the *x* direction. As the data points contain jitter because of non-uniform sampling, the FFT contained noise in the resulting spectrum, making it difficult to identify the spectral component with the maximum amplitude. To solve this issue, the measuring points were resampled to a regular grid with an increment size of the mean data point distance in the *x* direction. The resampling to the regular grid was carried out by linear interpolation between the points of the original measurement data. Afterwards, the FFT was calculated on this data set, using the corresponding built-in function of Octave [28]. Figure 11 shows the resulting spectrum for the grating, measured at a scanning speed of 10 μm $s^{-1}$.

In the spectrum above, the maximum amplitude at the nominal pitch can be clearly observed. Furthermore, there are additional peaks at the half, the third and the quarter of the nominal pitch. These peaks result from the rectangular shape of the profile and the resulting harmonics, as well as, e.g., an asymmetry in the profile. Lower frequencies/larger wavelengths indicate periodic distortions in the grating, mainly because of distortions during production.

As the measurement length is no integer multiple of the mean pitch, a shift in the maximum in the discrete spectrum results. This is known as the leakage effect [29] and poses a fundamental issue when using a Fast Fourier Transform for calculating the pitch. From the spectrum, the mean pitch over the complete scan length was calculated as 8.0033 μm. As described before, this value is resolution limited by the number of data points and the sampling rate. For the shown measurement, the pitch resolution is 1.28 nm. To overcome the resolution limitation, the pitch calculated by the FFT is used as a starting value in a Discrete Fourier Transform (DFT) based on the Goertzel algorithm [30]. The amplitude can be calculated for a spectral component of arbitrary frequency. Based on this initial value, a maximum search algorithm was started, using a Nelder–Mead approach [31] to find the maximum of the spectrum without any limitation in the resolution.

**Figure 12:**
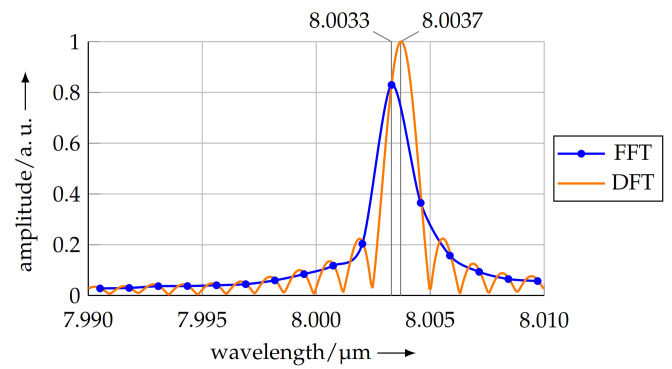
The resolution limitation of the FFT causes the maximum calculated amplitude not to be equal with the true maximum amplitude, visible in the DFT. However, the FFT maximum was used as initial parameter for the maximum search algorithm utilising a DFT.

Figure 12 shows a close-up view at the maximum of the FFT (see Figure 11) and the calculated peak frequency of the DFT.

The peaks in the figure above represent the calculated pitches of the FFT ($p_{FFT}$) and DFT ($p_{DFT}$) and are located at

(3) $$\begin{array}{cc} p_{FFT} & =8.0033\;\mu m\;\mathrm{and} \end{array}$$

(4) $$\begin{array}{cc} p_{DFT} & =8.0037\;\mu m\;. \end{array}$$

#### 4.3.3. Best-Fit Function

As the leakage effect leads to a slight shift in the maximum in the Fast Fourier transform, other methods incorporating the complete profile were investigated. One attempt is the approximation of the measured profile with an ideal, synthetic one. For this purpose, an ideal profile is numerically calculated and its parameters are optimised to best fit the measured profile in a least squares sense. To achieve this, the *z* values of an ideal square $z_{\text{squ,bf}}$ function as well as an ideal sine $z_{\text{sin,bf}}$ were calculated at the *x* positions of the measurement points.

(5) $$\begin{array}{cc} z_{\text{sin,bf}} & =A\;\sin\left(\frac{2\pi }{p_{\text{sin,bf}}}\left(x+\varphi_{\text{sin,bf}}\right)\right) \end{array}$$

(6) $$\begin{array}{cc} z_{\text{squ,bf}} & =A\;\mathrm{sgn}\;\sin\left(\frac{2\pi }{p_{\text{squ,bf}}}\left(x+\varphi_{\text{squ,bf}}\right)\right) \end{array}$$

Then, the period lengths ($p_{\text{sin,bf}}$, $p_{\text{squ,bf}}$) as well as the phase ($\varphi_{\text{sin,bf}}$, $\varphi_{\text{squ,bf}}$) of the two ideal functions were optimised to the measuring data by a Levenberg–Marquardt nonlinear regression [32]. The amplitudes (*A*) and offset of the ideal functions were fixed in this optimisation. The offset was set to zero, as the mean of the measuring data was subtracted from the scan line prior to the approximation. The amplitude was chosen to be equal to the standard deviation of the measuring points in the *z* direction.

**Figure 13:**
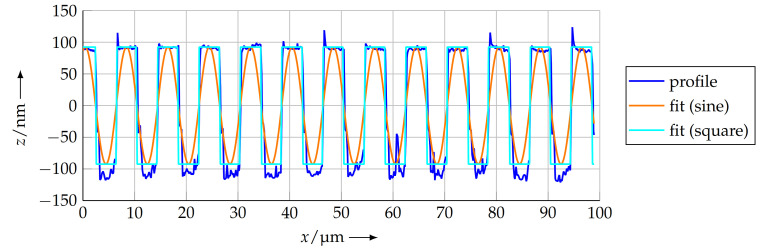
Best fit sine function and square function, tuned to the measured profile by minimising the squared error.

The initial parameters for the optimisation were taken from the COG method in Section 4.3.1 to ensure a proper approximation result. When any of the initial parameters for offset, amplitude, phase or period length differ too much from the real values, the optimisation will stop in a subsidiary maximum and thus result in a—fortunately obvious—incorrect approximation. After the optimisation finished, the parameters $p_{\text{sin,bf}}$ and $p_{\text{squ,bf}}$ were taken as the pitch of the measured profile. Figure 13 shows the profile as well as the two optimised functions.

For the profile measured at a scanning speed of 10 μm $s^{-1}$, the calculated pitches are

(7) $$\begin{array}{cc} p_{\text{sin,bf}} & =8.0038\;\mu m\;\mathrm{and} \end{array}$$

(8) $$\begin{array}{cc} p_{\text{squ,bf}} & =8.0038\;\mu m\;. \end{array}$$

#### 4.3.4. Lock-In Principle

The lock-in [33] principle, used as an additional evaluation method in this project, is similar to the best-fit method described before. Here, the basic idea is to determine the frequency with respect to the period of the measured data by comparison with a reference signal. For this purpose, the measured profile is multiplied point-wise with the values of the ideal reference profile. The resulting product is afterwards low-pass-filtered to obtain the amplitude at the current phase between measurement and reference profile. The low-pass filter chosen for this calculation is the mean over the complete length of the multiplication product. As in the previous section, the parameters of the reference profile were optimised to achieve a maximum mean amplitude over the complete scan length and, thus, the best overlay of the reference function to the measurement data. The reference profiles were once again calculated as an ideal square $z_{\text{squ,li}}$ function as well as an ideal sine $z_{\text{sin,li}}$, each with zero offset and an amplitude *A* equal to the *z* standard deviation of the measuring points.

(9) $$\begin{array}{cc} z_{\text{sin,li}} & =A\;\sin\left(\frac{2\pi }{p_{\text{sin,li}}}\left(x+\varphi_{\text{sin,li}}\right)\right) \end{array}$$

(10) $$\begin{array}{cc} z_{\text{squ,li}} & =A\;\mathrm{sgn}\;\sin\left(\frac{2\pi }{p_{\text{squ,li}}}\left(x+\varphi_{\text{squ,li}}\right)\right) \end{array}$$

**Figure 14:**
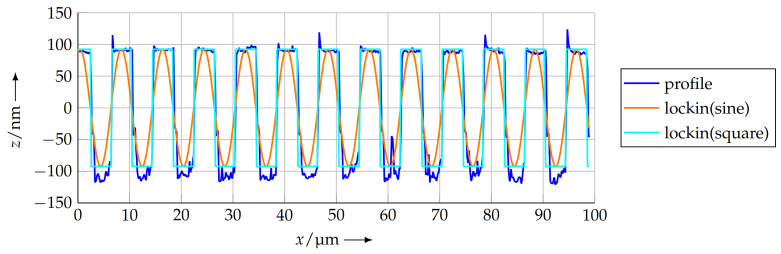
Approximated sine function and square function, tuned to the measured profile by a lock-in method.

The initial parameters for the optimisation were taken from the COG method and the parameters $p_{\text{sin,li}}$ and $p_{\text{squ,li}}$ were taken as the pitch of the measured profile. Figure 14 shows the profile as well as the two lock-in functions.

For the profile measured at a scanning speed of 10 μm $s^{-1}$, the calculated pitches are

(11) $$\begin{array}{cc} p_{\text{sin,li}} & =8.0038\;\mu m\;\mathrm{and} \end{array}$$

(12) $$\begin{array}{cc} p_{\text{squ,li}} & =8.0037\;\mu m\;. \end{array}$$

### 4.4. Comparison of the Results

The measurements and analyses described before were realised with a single grating sample, scanned on the same trace for maximum comparability. The six different scan velocities between 5 μm $s^{-1}$ and 50 μm $s^{-1}$ and the seven different analysis methods described in Section 4.3 result in a total of 42 values for the pitch of the grating under test. In this section, the results are compared to each other and analysed. Table 2 lists the values of the applied analysis methods for the different scan velocities. In the table, each column contains the mean of the pitch values obtained by the methods COG, DFT, best-fit (sine and square) and lock-in (sine and square). Values of the FFT are excluded from the respective mean calculation because of its limited resolution and the resulting—in general—large deviation from the other, unlimited, methods. The results in each single column are based on the same measuring data and should be equal in the ideal case.

Apparently, the measured pitch of the sample differs from the nominal pitch $p_{n}$ by approx. retain-explicit-plus +3.8 nm, which is a relative error of $+4.75\times 10^{-4}$, regardless of the evaluation method or scanning speed. This deviation can, in theory, be due to several reasons. The NFM-100 can be excluded to a large extent, because of the application of interferometric length measuring systems with stabilised He-Ne lasers (stability $10^{-8}\ldots 10^{-9}$) and an online correction of the refractive index of air (uncertainty $10^{-8}$) by the modified Edlén formula [34]. A misalignment of the sample also results in an error of the measured pitch. When the scan direction is not parallel to the grating direction, a cosine error arises, resulting in the measured pitch being too large. The rotation of the sample around the *z* axis and the resulting angle with respect to the *x* and *y* axes of the machine coordinate system is subject to future investigations (see Section 5). Another main factor is the thermal expansion of the linear scale made of glass, with a coefficient of thermal expansion of $8\times 10^{-6}$ $K^{-1}$. In general, the pitch error is caused by all three factors. As this paper focuses on the processing of the measurement data by different algorithms and their comparison, metrological investigations and different scan strategies will be the focus of future work.

**Figure 15:**
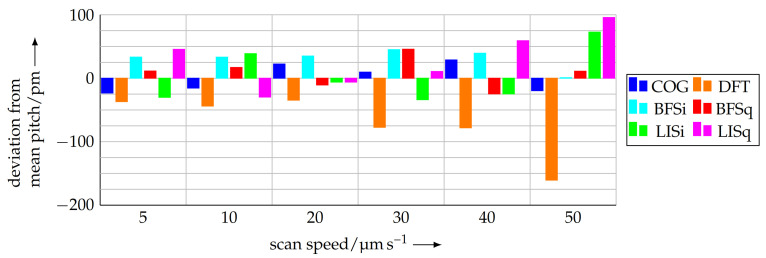
Deviations of the different analysis methods from the mean pitch for the according scan speed.

The differences in the analysis methods from the mean value for the according scan velocity are depicted in Figure 15. Here, it is observable that the deviation of the different evaluation methods between each other increases with the scanning speed. This is because of the increasing distortion of the measured profile due to overshoots, etc., the limited mechanical slew rate of the AFM and fewer data points per period. Furthermore, it is conspicuous that the pitch calculated with the DFT method is always lower than the mean value. The reason for this behaviour will be examined further in the project.

The differences between the evaluation methods are acceptable for scanning speeds in the range of 5 μm $s^{-1}$... 20 μm $s^{-1}$. The maximum deviation of retain-explicit-plus +45.6 pm for the lock-in method using a square function is equal to a length measuring error of only 0.29 nm over the complete scan length of 50 mm. Nonetheless, the measuring time is acceptable 42 min. The DFT apparently suffers from a decreasing number of data points per period with increasing scanning speeds (see Table 1).

The calculated standard deviations in Table 2 show that the COG method is the most reproducible analysis method for different scanning speeds and the resulting different point density per period. For the scanning speeds, a velocity of 20 μm $s^{-1}$ results in the smallest standard deviation, which is also observable in Figure 15. This medium speed seems to be a trade-off between lower speeds, suffering from drift effects because of the long measuring time, and higher speeds, causing overshoots and other distortions due to the mechanical properties of the tip-based system.

**Figure 16:**
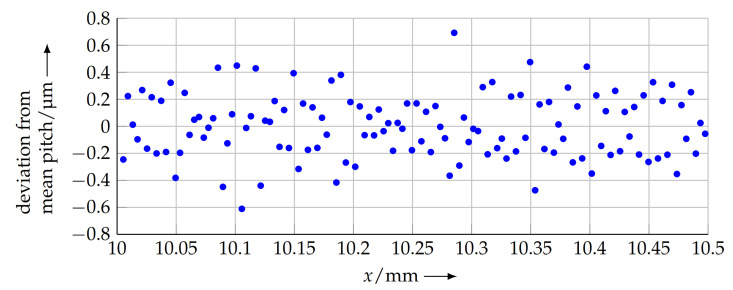
Per-period deviation from mean pitch (detail of complete scan line).

Figure 16 shows a detail of the calculated pitches for the complete scan length. Here, it is observable that each single period can be clearly distinguished and analysed. This opens up the possibility for a highly localised examination of the grating properties and the identification of possible manufacturing issues.

With these findings and the possibility of segmental evaluation, local pitch changes can be detected by using these high-precision measuring systems. This means that these local changes can be recognised again. As a result, statistical scales can be used in the future.

## 5. Conclusions and Outlook

The combination of the two presented measuring systems offers a novel possibility for the detection of defects as well as for quality control of large-area periodic grids such as diffraction gratings or length scales. With the help of the NFM-100, this can be realised up to an area of Ø100 mm with nanometre precision. In this paper, line scans over a length of 50 mm with a velocity up to 50 μm $s^{-1}$ are presented. These were evaluated using different approaches and finally compared with each other. It was shown that it is possible to reliably detect periods while excluding obvious profile errors (Section 4.3.1). By evaluating with different methods that take all measurement points into account, this provides a solid statistical base. In addition, the mentioned methods provide information about the local pitch of the measured sample and also the calculation of the mean pitch over the entire profile line. The machine table of the NFM-100 itself can achieve a standard deviation of 64 pm with respect to the ideal trajectory for the enormous length of 50 mm (Section 3).

In general, the described machine and methods can also be applied to two-dimensional scans. Here, the AFM will cover the *z* measurement, while the NFM-100 machine table will move in the *x* and *y* directions. In this way, several scan lines can be measured one beside another to gather the topography of an areal sample. In fact, this possibility of the NFM-100 will be exploited for more accurate measuring results in future investigations (see next paragraph). Furthermore, any other trajectory, be it circles, spirals or free forms, is also possible for the scan path, which might be useful for applications other than pitch measurements.

In the future, the focus will be on the segment-by-segment evaluation, with which it will be possible to apply the full-length analysis methods to local sections of the sample and thus the local accuracy of the sample to be analysed. This will make it possible to determine whether defects occur more frequently in certain areas. Furthermore, the dependence of the angle of the scan direction is important, which is why this should also be analysed and taken into account in more detail in the future, as was shown in [35], for example. This can be achieved by further investigation with different scan angles and several line scans next to each other. In addition, another focus is on the repeatability of the measurements, for which several line scans are necessary. Conclusions can then also be obtained about the tip wear of the microcantilevers. Long-range AFM scans are very time-consuming. Thus, the effect of temperature fluctuations cannot be ignored. To address the effect of thermal drift, additional investigations will be performed in the future. A possible method is to periodically interrupt a current measurement, go to a known reference position on the sample, determine the drift and then return to the previous position and resume the measurement.

## Figures and Tables

**Table 1:** Comparison of the sampling rate and the mean distance of the measuring points at different moving speeds. The period of the analyzed sample is 8 µm.

	Mean Sample Rate [Points/µm]	Mean Sample Rate [Points/Period]	Mean Point Distance [nm]
5 μm $s^{-1}$	12.2	97.5	82.1
10 μm $s^{-1}$	5.9	47.0	170.4
20 μm $s^{-1}$	3.0	23.7	337.9
30 μm $s^{-1}$	2.0	16.0	501.4
40 μm $s^{-1}$	1.5	12.0	666.8
50 μm $s^{-1}$	1.2	9.6	834.0

**Table 2:** Comparison of the calculated pitches for the different evaluation methods and the different scanning speeds. Values in a column should ideally be equal, as they are based on the same measuring data.

	Pitch/μm						
	Scanning Speed/μm $s^{-1}$						
Analysis Method	5	10	20	30	40	50	Stand. Dev.
COG	8.0038	8.0038	8.0038	8.0038	8.0038	8.0038	15 pm
DFT	8.0038	8.0037	8.0037	8.0037	8.0036	8.0036	54 pm
best fit sine	8.0038	8.0038	8.0038	8.0038	8.0038	8.0038	30 pm
best fit square	8.0038	8.0038	8.0037	8.0038	8.0037	8.0038	50 pm
lock-in sine	8.0039	8.0037	8.0037	8.0038	8.0038	8.0039	62 pm
Lock-in square	8.0039	8.0037	8.0037	8.0038	8.0038	8.0039	66 pm
mean	8.0038	8.0038	8.0037	8.0038	8.0037	8.0038	34 pm
standard deviation	35 pm	34 pm	25 pm	48 pm	51 pm	90 pm	
FFT	8.0034	8.0033	8.0032	8.0036	8.0038	8.0029	309 pm

## Data Availability

Research data are available upon request to the authors.
